# Euthanasia in the case of dementia: a survey among Flemish GPs

**DOI:** 10.3399/bjgpopen19X101677

**Published:** 2019-11-27

**Authors:** Jasper Cleemput, Birgitte Schoenmakers

**Affiliations:** 1 Master Thesis Student, Academic Centre of General Practice, Department of Public Health and Primary Care, University of Leuven, Leuven, Belgium; 2 Professor, Academic Centre of General Practice, Department of Public Health and Primary Care, University of Leuven, Leuven, Belgium

**Keywords:** general practice, euthanasia, dementia, Belgium, family practice, primary health care

## Abstract

**Background:**

In Belgium law prohibits euthanasia at the end stage of dementia when patients are no longer able to formulate their will. The number of applications for euthanasia based on dementia is low, but patients and their relatives are searching for access to euthanasia.

**Aim:**

This study assessed the opinions of GPs facing requests for euthanasia in patients with dementia.

**Design & setting:**

A cross-sectional survey was performed in general practice. Flemish GPs were invited by email to participate in the study.

**Method:**

GPs were reached through the regional GP association and the online survey was open for 4 weeks. The data were then anonymised, analysed, and interpreted. The outcome of interest addresses opinions of Flemish GPs regarding euthanasia in patients with dementia.

**Results:**

A total of 113/308 doctors participated. It was found that 69% agreed that euthanasia in patients without dementia is more acceptable than in patients with dementia. When patients with dementia had concomitant diseases, 59% stated that euthanasia was more acceptable than when patients were ‘healthy’. It was also found that 56% agreed the euthanasia law needs adjustments towards patients with dementia. Legal adjustments were supported more by GPs of a younger generation. Non-religious doctors were twice as likely to be in favour of legal adjustments than their religious colleagues; 51% believed that the ability of patients to repeat their will is essential; while 72% of GPs feared pressure from relatives to follow the declaration of will.

**Conclusion:**

The Belgian GP has an open attitude towards euthanasia for patients with dementia. There was a willingness to perform euthanasia as the stage of dementia worsened and in cases of terminal conditions. Debate, education, and experience will influence opinion and the legislation process.

## How this fits in

The debate on euthanasia in patients with dementia is ongoing in many countries. There is reluctance to perform euthanasia in cases where patients cannot repeat their will. There is a significant conflict between the aims of guaranteeing optimal end-of-life care and of respecting the wishes of a patient who cannot express their will.

## Introduction

In Belgium, as in many other countries, the law prohibits euthanasia when patients with dementia lack the cognitive capability to express their will. As these patients are no longer able to formulate a clear, voluntary, well-considered, and sustainable request for euthanasia, legislation does not allow the euthanasia procedure to be carried out in Belgium.^1^ Although the number of applications for euthanasia based on dementia is still low both in Belgium and in the Netherlands, patients and their relatives are increasingly searching for access to euthanasia.^2–4^

Initiating and fulfilling a request for euthanasia is a precarious and often loaded decision, not only for the patient and family, but also for the performing medical doctor. The GP’s response to a request depends on personal, ethical, and psychological arguments, as well as legal conditions.^5^ The view of GPs on euthanasia for physically or mentally suffering patients is well researched, but very little is known about GPs’ experiences and opinions when it comes to euthanasia in the case of dementia.^6^

In the Netherlands, it recently (2016) became possible to carry out euthanasia under strict conditions for patients with advanced dementia. Two studies showed that almost 90% of the general population surveyed in the Netherlands found euthanasia in advanced dementia acceptable , while only 30-40% of GPs were willing to cooperate in a euthanasia request in a patient with dementia.^3,5,7^ Research among Belgian psychiatrists showed that over three-quarters of the doctors would agree to carry out euthanasia at a late stage of dementia, based on a recent advance directive.^8,9^ Other research showed that religious doctors were more reluctant to perform euthanasia than non-religious doctors, and that doctors with more years of work experience were more receptive to this idea compared with their younger colleagues with less work experience.^9,10^

Today in Belgium, the controversial discussion on euthanasia in patients with dementia is high on the agenda of policymakers, patient groups, and professional caregivers. Therefore, this study assessed the opinions of Flemish GPs about the practice of euthanasia in patients with dementia.

## Method

A cross-sectional survey was performed according to the computer-assisted web interviewing (CAWI) method. It was conducted via email with a reminder after 2 weeks. Flemish GPs were invited to participate in the study and to anonymously complete an online questionnaire. GPs (*n* = 308) were reached through the regional GP association. The questionnaire assessed the opinions of the physician regarding the request for euthanasia in people with dementia. To set the baseline of a good understanding of the term ‘euthanasia’, the questionnaire was introduced with an explicit referral to the formal, legal definition. The outcome of interest was determined as opinions of Flemish GPs regarding euthanasia in patients with dementia under the current legislation. The secondary outcome was determined as the opinions of Flemish GPs regarding euthanasia in general.

The questionnaire was based on existing questionnaires with the addition of questions specifically targeting the research question.^3,11^ There were nine questions about the doctor's characteristics: two open questions, two multiple-choice questions, and five yes or no questions. Six questions addressed the general idea of ​​euthanasia and were answered with a 5-point Likert scale (from ‘strongly agree’ to ‘strongly disagree’). Twelve questions addressed views on ​​euthanasia in a patient with dementia, 11 of which were answered with a 5-point Likert scale (from ‘strongly agree’ to ‘strongly disagree’), and one with ‘yes’ or ‘no’ and a free-text field. Free-text answers were analysed following the Grounded Theory of Glaser and Strauss.^12^

After 4 weeks, the survey was closed; the data were anonymised, analysed, and interpreted.

Analysis were uni- and bivariate and processed by Excel (version 2016 Professional plus). In bivariate analyses, a two sample *t*-test was used and *P* values were calculated.

## Results

A total of 113 doctors completed the survey fully, which represents a response rate of 37 %.

Characteristics of participating GPs are displayed in Table 1. Based on age, three categories were made: 42% (*n* = 48/113) of all GPs were aged 26–40 years (category 1); 20% (*n* = 23/113) were aged 41–55 years (category 2); and 37% (*n* = 42/113) were aged ≥56 years (category 3). Male (53% or *n* = 60/113) and female (47% or *n* = 53/113) GPs were approximately equally represented. The average working experience was 19 years.

**Table 1.** Characteristics of participating GPs

**Characteristic**	***n* (%**)
**Age** **,** **years**	
26–40	48 (42)
41–55	23 (20)
≥56	42 (37)
**Sex**	
Male	60 (53)
Female	53 (47)
**Religious**	
Yes	46 (41)
No	67 (59)
**Ha** **s** **or** **had a family member with dementia**	
Yes	63 (56)
No	50 (44)
**Has performed euthanasia in the past**	
Yes	71 (63)
No	42 (37)
**Has previously performed euthanasia in patient with psychological suffering**	
Yes	24 (21)
No	89 (79)

The results showed that 41% (*n* = 46/113) of all GP has described themselves as being ‘religious’, in the broad meaning of the word, the majority of whom were aged >40 years (74%). Sixty-three per cent (*n* = 71/113) stated they had performed euthanasia in the past, and 21% (*n* = 24/113) stated they had already performed euthanasia in the case of a patient who was mentally suffering. Fifty-six per cent (*n* = 63/113) of GPs indicated they have or have had a family member with dementia.

It was found that 52% (*n* = 59) of all responders agreed that euthanasia in patients without dementia felt more acceptable than in patients with dementia. When patients with dementia suffered one or more concomitant diseases, 45% (*n* = 51) of all responders stated that euthanasia was more acceptable than when the dementia patient was overall ‘healthy’. When asked whether a terminal condition was a necessary criterion to perform euthanasia, 48% (*n* = 54/113) of all GPs disagreed.

**Figure 1.**
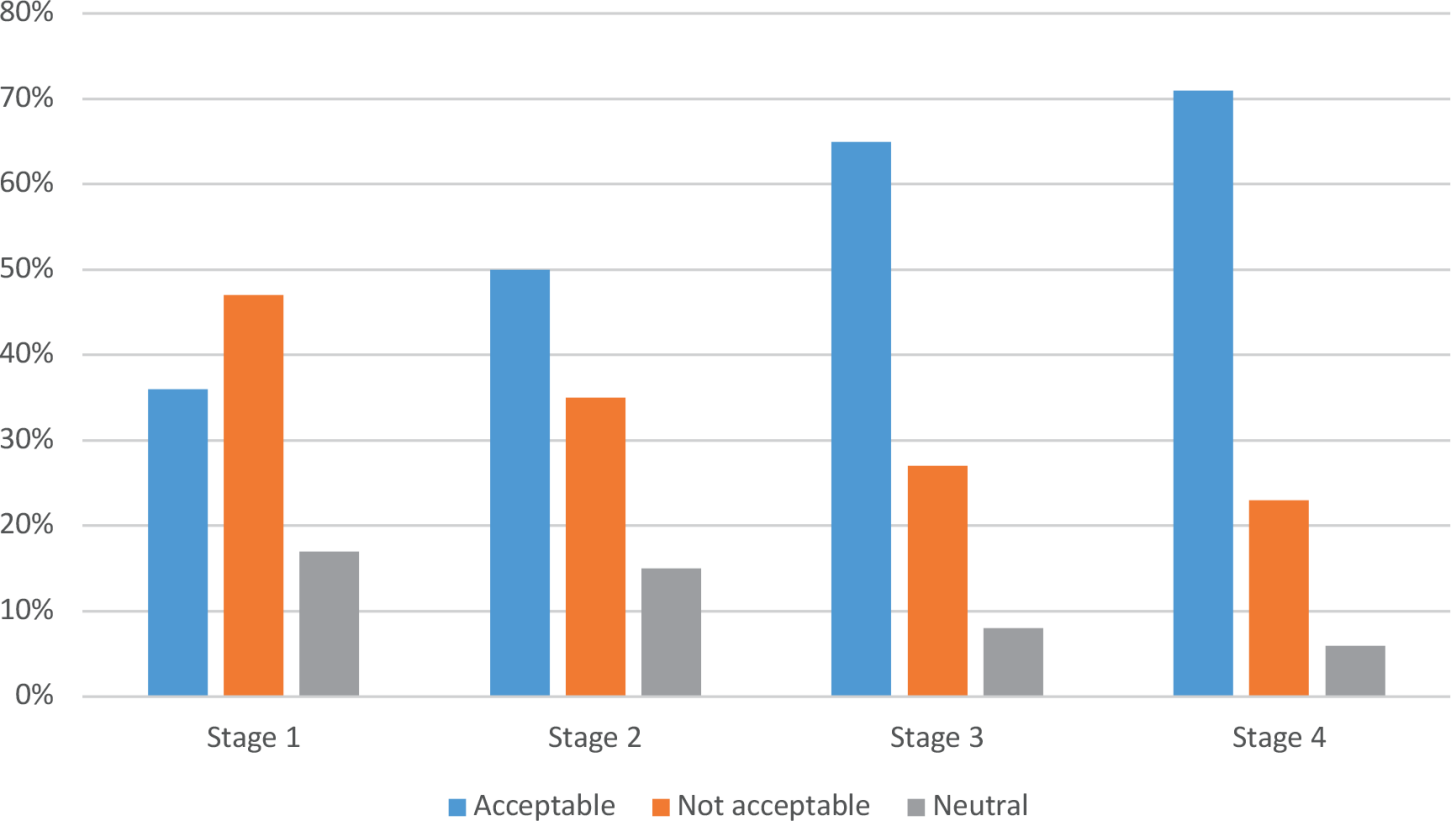
Acceptability of performing euthanasia at different stages of dementia. Stage 1 = early dementia (moments of forgetfulness, fear of what will come, and sickness awareness); stage 2 = progressive dementia (disorientation, reduced awareness of time, increasingly worse recognition of loved ones); stage 3 = advanced dementia (complete withdrawal, without any sense of time, place, or person, endlessly engaged in certain movements and/or sounds); stage 4 = end-stage dementia (contactless, hardly any reaction, only physically present).

When asked at which stage of dementia the performing of euthanasia, based on an advance directive, was acceptable for the GPs, a rising trend was seen as the stage of dementia progressed (Figure 1).

Non-religious doctors were more willing to perform euthanasia in all stages of dementia compared with religious doctors, but the difference was more pronounced in the early stage (50% [*n* = 34/67] versus 15% [*n* = 7/46]) and in the end-stage of dementia (81% [*n* = 54/67] versus 56% [*n* = 26/46]).

When asked if the euthanasia law needs adjustment with regards patients with dementia, only 43% (*n* = 49/113) agreed (Table 2). Further, 66% (*n* = 75/113) of all physicians indicated that the current euthanasia legislation in Belgium does not provide room for assistance with a request for life-ending support in patients with dementia. Legal adjustments were encouraged more by the younger generation GPs compared with GPs aged >40 years (63% [*n* = 30/48] versus 48% [*n* = 31/65]; *P*<0.005). Non-religious doctors were almost twice as likely to be in favour of legal adjustments in comparison with religious doctors (66% [*n* = 44/67] versus 37% [*n* = 17/46]; *P*<0.001).

**Table 2.** Participant responses to statements regarding dementia and euthanasia (5-point Likert scale)

Statement	‘Rather not’ to ‘not agree’, *n* (%)	‘Rather agree’ to ‘completely agree’, *n* (%)
Euthanasia in patients without dementia is more acceptable than in patients with dementia	17 (19)	59 (69)
Euthanasia in patients with dementia and concomitant diseases is more acceptable than in cases of 'healthy' dementia patients	21 (24)	51 (59)
A medically classifiable condition (that is, more than a medical basis) is a requirement to perform euthanasia or assisted suicide	29 (33)	40 (43)
In an older person, who suffers unbearably due to an accumulation of medical and non-medical problems, euthanasia or medically assisted suicide may be acceptable to me	18 (20)	63 (70)
Euthanasia or medically assisted suicide is only acceptable to me if the patient suffers from a terminal illness	54 (59)	28 (30)
At the first^a^ stage of dementia, euthanasia is acceptable	40 (46)	32 (44)
At the progressive^b^ stage of dementia, euthanasia is acceptable	22 (39)	44 (51)
At the progressed^c^ stage of dementia, euthanasia is acceptable	25 (29)	56 (65)
At the final^d^ stage of dementia, euthanasia is acceptable	21 (24)	63 (72)
Legislation on euthanasia in patients with dementia needs adjustment	20 (34)	49 (56)
The current euthanasia law offers no room for assistance with a request for termination of life in patients with dementia	8 (9)	75 (85)
With regards to legislation of euthanasia, dementia and coma patients are to be treated equally	24 (28)	58 (67)
It is important that the patient gives their consent at the decisive moment	26 (30)	44 (51)
If legislation allows euthanasia in case of dementia, relatives will put pressure on at the decisive moment	14 (16)	63 (72)

Category 'neutral’: omitted. ^a^Moments of memory loss, fear of future, aware of disease. ^b^Disorientation in time and space, problems recognising relatives. ^c^Regression, no awareness of time and space, repetition of actions and noises. ^d^No interaction, no communication, no active reactions.

When asked if the legislation on euthanasia in patients in comas should be expanded to all patients (including patients with dementia), 72% (*n* = 63) of all GPs, and 77% (*n* = 37/48) of GPs aged <41 years, agreed. Religious doctors were more than twice as likely to disagree with this statement as non-religious doctors (39% [*n* = 18/46] versus 18% [*n* = 12/67]; *P*<0.001). It was found that 51% (*n* = 44) of the surveyed doctors believed that the ability of patients to formulate their will at the decisive moment of euthanasia is essential (Table 2).

If legislation allowed euthanasia, confirmed by an advance directive in patients with dementia, 72% (*n* = 63) of GPs feared the pressure of relatives to follow the declaration of will without the explicit expression of consent by the patient at the decisive moment (Table 2).

In the last question of the survey, GPs were asked whether they would consider carrying out euthanasia in a patient with end-stage dementia, supported by an advance directive. It was found that 62% (*n* = 70/113) considered doing this, 33% (*n* = 37/113) answered that they would not, and 5% (*n* = 6/113) doctors answered they did not know.

From the free-text comments in answer to ‘If legislation allows euthanasia in later than the beginning stages of dementia, would you carry out euthanasia (if advance directive available)?’, it was learnt that 41% (*n* = 36/86) of all responders (unconditionally) had a positive attitude towards euthanasia in cases of dementia, while 20%^13^ would never consider this action (Table 3). Twelve per cent^10^ of the GPs explicitly insisted on a shared decision with patients, relatives, and carers (professional and informal; Table 3).

**Table 3.** Free-text comments to question: 'If legislation allows euthanasia in later than the beginning stages of dementia, would you carry out euthanasia (if advance directive available)?'

**Answer label**	***n* = 86 (completed answers)**
Yes (if legal)	36
No, never	18
Only if complicated with physical comorbidities or other threatening conditions	3
No, since you cannot judge quality of life and meaning to others	2
No, since I want a clear ‘yes’ from the patient	5
Yes, if patient, family, relatives, and other carers are extensively involved and consulted in a shared decision-making	11
Yes, but considering that an advance directive does not necessarily reflect today's beliefs or wishes and that dementia was specified in the directive	4
Yes, if you know the patient, their life lifecycle, and their life view very well	4
No opinion	3

## Discussion

### Summary

A two-third majority of physicians believed that euthanasia in patients without dementia is more acceptable than in patients with dementia. In particular, religious doctors are more reluctant to perform euthanasia. Over half of the participants believed that the performance of euthanasia is more acceptable in cases of serious concomitant disease or of terminal disease than in the absence of these conditions.

### Strengths and limitations

This study ran at a moment of intense discussion about euthanasia in the case of dementia in Belgium. Participating doctors showed a lot of interest in the topic and responded comprehensively to the open question. These answers and opinions were largely in agreement with other research on this topic.

It is plausible that mainly physicians with experience or affinity with the discussion on euthanasia in patients with dementia participated in this study.^12^ On the other hand, the relative participation of adherents and opponents was comparable with participation in other studies.

A weakness of the study is the relatively small number of participants. However, as compared with the general GP population, the sample was representative with respect to age, sex, and performance of euthanasia in the past.^14,15^

### Comparison with existing literature

As demonstrated in previous studies, medical doctors in general and GPs in particular share feelings of restraint towards euthanasia in patients with dementia. These feelings are also reflected in the observation that only half of the GPs are in favour of legal adjustments giving access to euthanasia in cases of dementia. In contrast, three-quarters of the GPs state that euthanasia in patients with end-stage dementia should be approached in the same way as with patients in comas. Doctors apparently still adhere to a biomedical model of health care, wherein a medical condition plays a decisive role in the care process.^1,5,11^

This study showed that as dementia worsens, more doctors find performing euthanasia acceptable; in contrast, not even half of the practitioners would perform euthanasia at the early stages of dementia. Suffering a terminal illness remains an important condition to granting a euthanasia request.^3,16,17^ Two-thirds of all GPs would perform euthanasia in a patient with end-stage dementia if that patient had a clear declaration of will and if legislation is adjusted to this condition. Arguments to support these statements include respect for the patient's autonomy, dignity, and quality of life.^11,18^ GPs in this study also mention that the option for euthanasia in the late stage of dementia raises feelings of reassurance and relief in patients and relatives. On the other hand, GPs fear the influence of relatives at the decisive moment of euthanasia. This fear was also expressed by 91% of doctors in a Dutch study.^3,19^ At the same time, some doctors put forward the option to consult and involve relatives at the decisive moment to perform a euthanasia request.^4,9^

Doctors who disagree on euthanasia as an option in the late stage of dementia argue that it is impossible to judge the quality of life in these patients and that, therefore, the patient must always be able to repeat their will at the decisive moment. This argument emphasises the importance doctors assign to the autonomy of a patient and to sharing the decision process.^5,6,18^ Hence, this is also an expression of the conflict between meeting the right of self-determination of the patient and the duty of a doctor to guarantee the quality of the care process at the end of life.

It also appears that certain demographic characteristics of the responders (age, religious versus non-religious, having a family member with dementia) affect the responses. First, the number of doctors considering themselves as ‘religious’ was higher than expected according to the general population (41% versus 28%). It is not known whether doctors are traditionally more religious or whether there has been a participation bias, but the same numbers were found in another publication.^20^ A more conservative attitude was consistently noticed towards euthanasia in the case of religious doctors compared with their non-religious colleagues, as also demonstrated in other studies.^13,20^

Second, it was noticed that age or years of working play an important role. Younger physicians show the most open attitude towards euthanasia in dementia. This might be explained by an intergenerational difference in norms and values, and with the fact that younger doctors grew up with the debate on euthanasia.^5,7–10,21^ Younger doctors also lack experience and therefore may not have been confronted with difficult and delicate situations.^9^

It is noteworthy that doctors aged 41–55 years answered the questions more conservatively compared with both the older and the younger generation. This trend was also observed in other studies.^3,9,16^ A possible explanation for the openness of the oldest group of doctors towards euthanasia is that these doctors already faced concrete situations with dementia and euthanasia requests, possibly even within their own family.^10^

The barrier to performing euthanasia in the case of dementia seems to be lower for GPs who have or have had a family member with dementia than it is for doctors without a family member with dementia. Their experience with these ill relatives likely plays a role in this observation.^16^ The older doctors are also entering an age group where they start to reflect on the situation in relation to their future selves.^16^

When comparing the results to the Dutch studies, there is a trend that is worth mentioning: where the acceptability of euthanasia increases with the progression of the dementia for Belgian GPs, it *decreases* under the same conditions for Dutch GPs.^3,5^ Another important finding is that, where the present study shows that 85% of all GPs believe the current euthanasia legislation in Belgium does not provide room for euthanasia in patients with dementia, only 38% of Dutch GPs believe this to be the case with their national euthanasia legislation. Legislation in both countries is, however, very similar (at the time of this research). It seems that the view of Flemish and Dutch GPs differs largely on this issue. This observation also might be influenced by the fact that the legal requirements and conditions to perform euthanasia are more complicated in Belgium compared with the Netherlands. Different interest groups in both countries, however, dominate the public and professional debate on euthanasia.^3,5,13,22^

### Implications for research and practice

In conclusion, the Belgian GP has an open attitude towards euthanasia for patients with dementia. A rising trend of willingness to perform euthanasia was found as the stage of dementia worsened and in cases of terminal conditions or serious concomitant diseases. Given the finding that the youngest, the oldest, and non-religious doctors appear to have more openness towards euthanasia for patients with dementia, debate, education, and experience will influence the opinion and the legislation process. Research should focus on the underlying conditions and characteristics that influence medical doctors when facing a euthanasia request in patients with dementia.

## Dataset

The complete dataset is available on simple request and will be sent as a link to a google drive directory.
